# Non-Invasive Targeted Hepatic Irradiation and SPECT/CT Functional Imaging to Study Radiation-Induced Liver Damage in Small Animal Models

**DOI:** 10.3390/cancers11111796

**Published:** 2019-11-15

**Authors:** Rafi Kabarriti, N. Patrik Brodin, Hillary Yaffe, Mark Barahman, Wade R. Koba, Laibin Liu, Patrik Asp, Wolfgang A. Tomé, Chandan Guha

**Affiliations:** 1 Department of Radiation Oncology, Institute for Onco-Physics, Albert Einstein College of Medicine, Bronx, NY 10461, USA; rkabarri@montefiore.org (R.K.); nibrodin@montefiore.org (N.P.B.); Hillary.Yaffe@einstein.yu.edu (H.Y.); mark.barahman@med.einstein.yu.edu (M.B.); wade.koba@einstein.yu.edu (W.R.K.); laibin.liu@einstein.yu.edu (L.L.); patrik.asp@einstein.yu.edu (P.A.); wtome@montefiore.org (W.A.T.); 2 Department of Radiation Oncology, Montefiore Medical Center, Bronx, NY 10461, USA; 3 Department of Surgery, Albert Einstein College of Medicine, Bronx, NY 10461, USA; 4 Department of Pathology, Albert Einstein College of Medicine, Bronx, NY 10461, USA; 5 Department of Urology, Albert Einstein College of Medicine, Bronx, NY 10461, USA

**Keywords:** liver irradiation, radiation-induced liver disease, small animal radiation research platform, SPECT/CT imaging

## Abstract

Radiation therapy (RT) has traditionally not been widely used in the management of hepatic malignancies for fear of toxicity in the form of radiation-induced liver disease (RILD). Pre-clinical hepatic irradiation models can provide clinicians with better understanding of the radiation tolerance of the liver, which in turn may lead to the development of more effective cancer treatments. Previous models of hepatic irradiation are limited by either invasive laparotomy procedures, or the need to irradiate the whole or large parts of the liver using external skin markers. In the setting of modern-day radiation oncology, a truly translational animal model would require the ability to deliver RT to specific parts of the liver, through non-invasive image guidance methods. To this end, we developed a targeted hepatic irradiation model on the Small Animal Radiation Research Platform (SARRP) using contrast-enhanced cone-beam computed tomography image guidance. Using this model, we showed evidence of the early development of region-specific RILD through functional single photon emission computed tomography (SPECT) imaging.

## 1. Introduction

The incidence of liver cancer is currently increasing in the United States and represents one of the most common malignancies worldwide, with deaths related to liver cancer expected to surpass those of breast, prostate, and colorectal cancer within the next few decades [1]. Although radiation therapy (RT) is used in one third of all cancer patients, the role of RT in hepatic malignancy has traditionally been limited by the presumed low radiation tolerance of the liver, after early studies demonstrated whole liver radiation in excess of 30–35 Gy to be associated with a high risk of radiation induced liver disease (RILD) [2,3,4]. The recently published Quantitative Normal Tissue Effects in The Clinic (QUANTEC) report on radiation-associated liver injury confirmed that the risk of RILD in the treatment of primary liver tumors increases rapidly as the mean liver dose becomes greater than 30 Gy in 2-Gy fractions [5]. Previous work in our laboratory has established animal models of radiation-induced liver damage in rats, although with the need for RT to be delivered via laparotomy to deliver high doses of radiation [6,7]. Intra-operative procedures for delivering liver RT are however not ideal for studying radiation-induced liver damage in a translational setting as this is not in agreement with how patients are treated for liver cancer. Other studies have used clinical linear accelerators to deliver whole- or partial-liver RT to rats by imaging them on a computed tomography (CT) scanner and marking the extent of the liver on the skin of the animal [8,9]. Although an improvement, this technique still restricts the delivery of partial-liver RT to rats due to size restrictions, and to using external skin markings to guide the field setup. Additionally, the dose that can be delivered using this technique is limited by dose to bowel. Patients with liver tumors are typically treated with regional high-dose RT, often using highly conformal treatment techniques, to only the part of the liver that contains the tumor. Performing regional liver RT in a small animal model, which would thus be more translationally accurate, is technically challenging especially for mice due to the limited size of their liver. However, the recent development of dedicated robotic platforms such as the Small Animal Radiation Research Platform (SARRP, Xstrahl, Surrey, UK) has made image-guided targeted RT feasible for rodent models [10,11].

In humans, RILD is manifested as hepatic sinusoidal obstruction syndrome (SOS) along with hepatic central venous occlusive disease (VOD). Although the onset of hepatic VOD is not seen in rodents after whole-liver RT, electron microscopy can show endothelial cell death and dehiscence at 24 h after liver RT. In addition, TUNEL-staining demonstrated that apoptosis is induced in liver sinusoidal endothelial cells (LSEC), within 6 h of liver irradiation [12].

Taken together, this led us to develop a non-invasive technique for delivering targeted regional liver RT to mice using the SARRP and combined Single Photon Emission CT and CT (SPECT/CT) functional imaging to characterize the early changes and pathogenesis of RILD.

## 2. Materials and Methods

### 2.1. Animal Model and Hepatic Irradiation Technique

Hepatic irradiation was delivered to either male C57Bl/6, male Rag2^−/−^γ(c)^−/−^ mice obtained from NCI (Fort Dietrich, MD, USA), Cirrhotic DPPIV deficient C57Bl/6 mice (Special Animals Core of the Marion Bessin Liver Research Center) at 6–8 weeks of age, to examine models of varying radiosensitivity. Animals were housed in the Institute for Animal Studies and all animal handling and irradiation procedures were performed in accordance with an animal protocol approved by the Institutional Animal Care and Use Committee at the Albert Einstein College of Medicine (Protocol number 20171207; approved on 22 March 2018). Cirrhosis in DPPIV deficient C57Bl/6 mice was induced by using CCl_4_ administration (intraperitoneal [IP] injections twice a week for 11 weeks) [13,14]. Prior to RT delivery the animals received 100 µL ExiTron nano 6000 liver contrast enhancement agent (Miltenyi Biotec Inc., San Diego, CA, USA) via lateral tail vein injection, corresponding to a dose equivalent to 640 mg iodine/kg body weight (for a 25 g mouse).

Liver imaging was performed 24 h after injection in order to easily identify the liver using the on-board cone-beam CT (CBCT) imaging capabilities of the SARRP. The animals also received 3% Gastrografin contrast solution (Bracco Diagnostics Inc., Cranbury Township, NJ, USA) delivered orally through gastric lavage to visualize the stomach and bowels, so that we could ensure that no part of the gastrointestinal (GI) tract received a radiation dose of more than 17 Gy, which was sufficient to avoid any GI syndrome in these animals.

**Figure 1:**
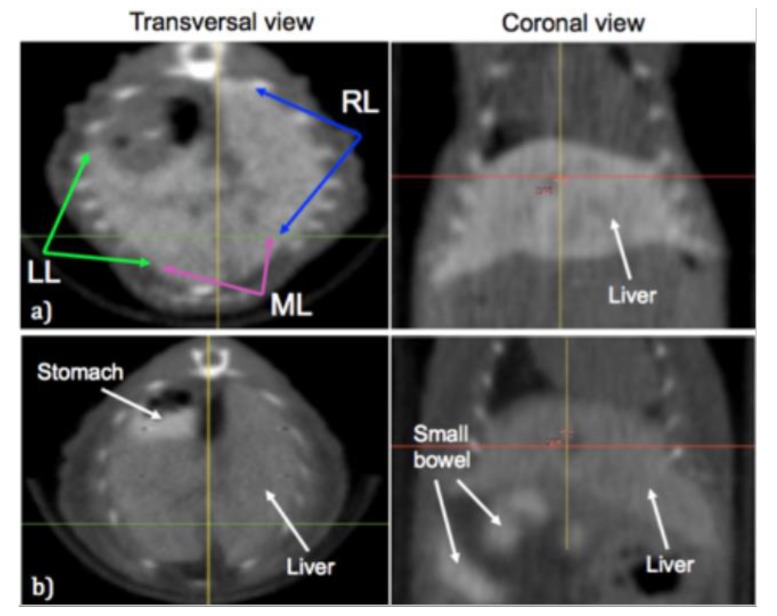
Contrast-enhanced liver imaging. Transversal and coronal cone-beam computed tomography images are shown for a mouse that was administered liver contrast agent in (**a**) with the left lobe (LL), median lobe (ML) and right lobe (RL) indicated. In (**b**) the images show a mouse that was administered both the liver contrast agent and the gastrointestinal contrast agent, highlighting the stomach and small bowel.

Following administration of oral contrast, the animals were anesthetized using a continuous flow of 1.5% Isoflurane in 1.5 liters/minute pure oxygen. To deliver the targeted irradiation, the animals were placed one at a time on the SARRP stage and a CBCT scan was performed using 50 kVp and 0.7 mA scan settings. The individual lobes of the liver and any nearby stomach or bowel was then identified on the CBCT scans as shown in Figure 1.

**Figure 2:**
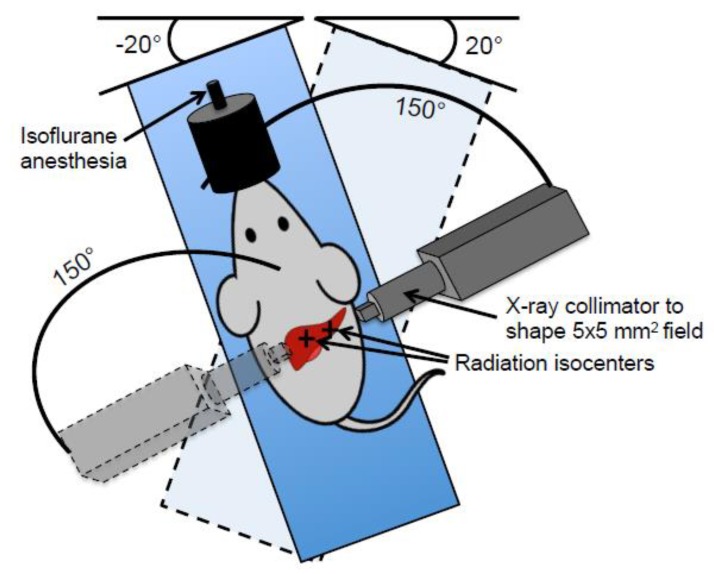
Illustration depicting targeted liver irradiation. The irradiation field setup is illustrated showing the partial arc radiation delivery at either a 20° or −20° stage rotation. The relative size of the animal is exaggerated as compared to the X-ray collimator for visualization purposes.

The targeted liver irradiation was then specified in the treatment planning system of the SARRP with the aim of delivering 50 Gy to a 5 × 5 mm^2^ area of the median lobe and 25 Gy to a 5 × 5 mm^2^ area of the right lobe, while leaving the caudate lobe and inferior left lobe untreated and keeping the maximum dose to any part of the GI tract below 17 Gy. This was achieved by administering the radiation as two partial arcs of 150° each with a 20° stage rotation, or “couch kick”, in opposite directions for each of the two isocenters, as illustrated in Figure 2.

**Figure 3:**
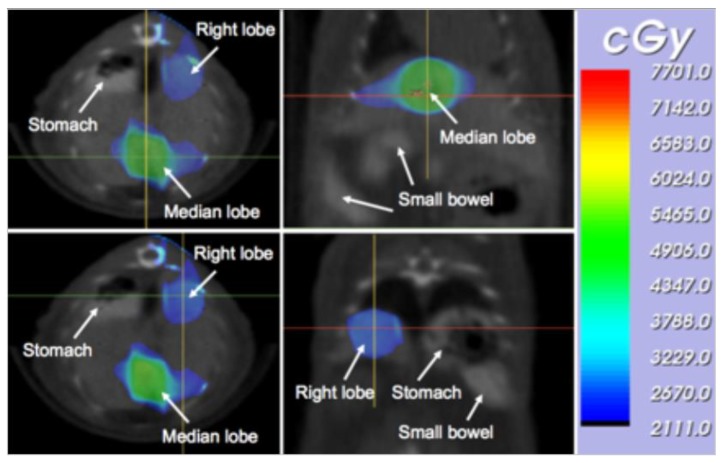
Dose color-wash depicting radiation isocenter setup. The calculated radiation dose distribution is shown as a color-wash for two isocenters delivering 50 Gy to the median lobe and 25 Gy to the right lobe, with the dose given as cGy (1 Gy = 100 cGy). It should be noted that the incident irradiation fields were placed so that the spinal cord of the animal was spared from direct high-dose irradiation to avoid causing hind limb paralysis in the animals.

The irradiation time was computed individually for each animal based on the desired dose at the median lobe isocenter and right lobe isocenter. An example of the resulting dose distribution is shown in Figure 3 for the two radiation isocenters.

Following the treatment planning and dose calculation, the targeted liver RT was delivered using a 5 × 5 mm^2^ square collimator and 220 kVp X-ray energy with 13 mA tube current and a 0.15 mm Cu filter. The total time to deliver the prescribed radiation dose to both isocenters within the liver was about 30 minutes per animal once the CBCT imaging and treatment planning was completed, at a dose rate of ~2.3 Gy/min.

### 2.2. Functional Imaging to Assess Radiation-Induced Liver Disease

Liver function was assessed in vivo at 2 months after irradiation to assess functional indications radiation-induced liver damage in the irradiated lobes. Functional imaging was performed using the Siemens Inveon^TM^ small animal micro SPECT/CT platform (Siemens AG, Munich, Germany). We used ^99^Tc^m^-labelled Sulfur Colloid as the radioactive tracer since sulfur colloid uptake by the Kupffer cells reflects the perfusion and functionality of liver cells and thus gives a measure of regional functionality within the liver [15] Additionally, we have previously shown that hepatic irradiation suppressed the phagocytic function of Kupffer cells injected with colloidal carbon in animals [12]. The tracer was administered through retro-orbital intra venous injection at an activity of 0.5–0.6 mCi, immediately prior to image acquisition. The animals were anesthetized using 1.5% Isoflurane in 1.5 liters/min pure oxygen during injection and imaging procedures, and the animals were injected inside the SPECT/CT platform to allow for immediate imaging. SPECT scans were acquired using the “5-MWB-1.0” mouse whole-body 5 pinhole collimator.

SPECT data was acquired from 48 separate projection angles spanning 180° with 3.7° spacing, with 20 s acquisition time at each projection angle and reconstructed using an OSEM-3D algorithm with 8 iterations and 4 subsets. Lastly, the CT scans were acquired without changing the animal position using a setting of 80 kVp, 0.5 mA and 200 ms exposure time per projection angle, with 600 s total acquisition time and 600 s reconstruction time with the same OSEM-3D protocol.

### 2.3. Histological Analysis of Irradiated Liver Samples

Histological staining was performed on irradiated mouse livers at 2 h after the delivery of targeted RT. Animals were sacrificed and livers were excised, fixed in formalin and embedded in paraffin followed by γH2AX antibody (Sigma-Aldrich, St. Louis, MO, USA) staining as a marker of DNA damage resulting from double strand breaks. Sections of 5 μm thickness were used and imaged at 4× and 20× magnification following γH2AX staining.

## 3. Results

The irradiation procedure was well tolerated by all animals and no subjects were lost to acute GI syndrome or died from any other causes, showing that limiting the radiation dose to the GI tract to <17 Gy through Gastrografin visualization was sufficient. Animals were kept in a heated recovery cage following the radiation procedure and returned to normal activity level within 10 minutes after being taken off anesthesia. A minimum of three animals were used for each study condition when developing this model.

**Figure 4:**
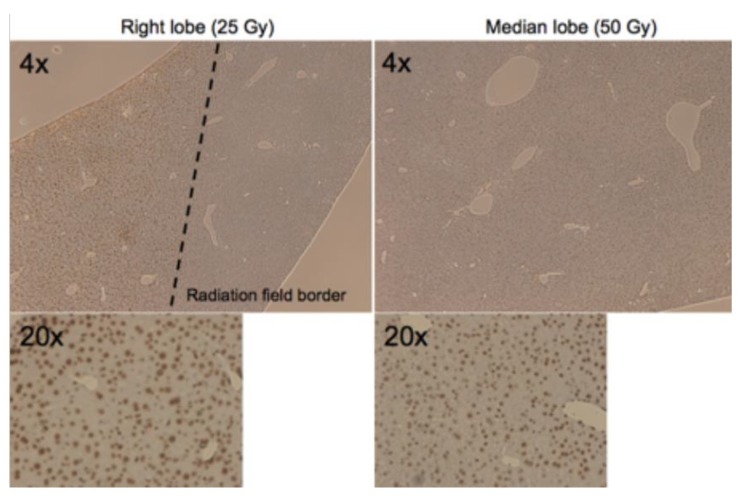
Histopathological staining shows demarcation of DNA damage within radiation field border. Histopathological γH2AX staining of sections from the right and median of an animal that received the targeted liver irradiation. The sections are shown in lower magnification (4×) as well as high-power (20×) magnification, with γH2AX-positive cells appearing in dark brown color, indicating double strand break DNA damage. The dashed line in the right lobe section shows the clear dichotomization between irradiated and un-irradiated liver tissue.

A sub-group of animals were sacrificed at 2 h post irradiation and the liver tissue was analyzed for DNA damage using γH2AX-staining, and Figure 4 shows the resulting staining for the median and right lobe of one of the irradiated animals. The histopathological staining shows clear and widespread double strand break DNA damage in the irradiated lobes, in the lobe that received 25 Gy and the one that received 50 Gy. It also showed a dramatic difference between irradiated and un-irradiated liver tissue, with virtually no γH2AX-positive cells in the un-irradiated tissue, consistent with the sharp penumbra of the targeted irradiation field.

**Figure 5:**
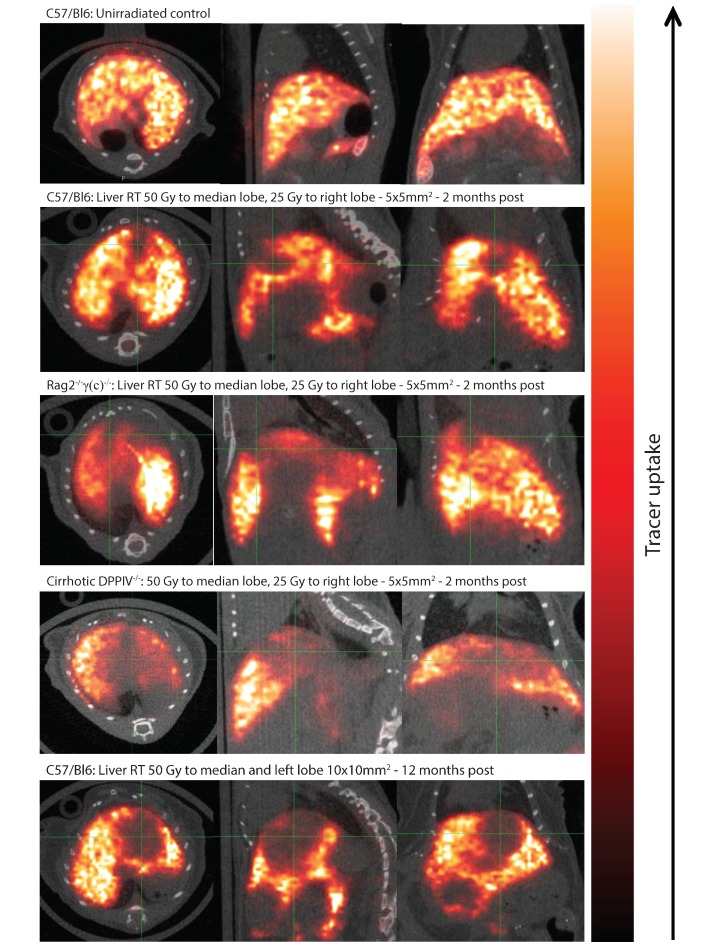
Functional SPECT/CT imaging shows reduced Kupffer cell perfusion in irradiated areas. The SPECT/CT images were taken at respectively 2 months and 1 year post targeted liver irradiation with the CT showing the underlying anatomy and the overlaying color-wash shows the uptake of ^99^Tc^m^-labeled Sulfur Colloid within the liver. The bottom three panels show irradiatd livers with substantially reduced uptake in the areas that received high-dose irradiation lobe, indicative of reduced Kupffer cell perfusion following irradiation. The top panel shows an age-matched control animal that did not receive any liver irradiation.

At 2 months post irradiation another group of animals were assessed for region-specific liver function using the ^99^Tc^m^-labeled Sulfur Colloid SPECT/CT imaging protocol. Figure 5 shows the region-specific tracer uptake as a marker of functioning Kupffer cells within the different parts of the irradiated liver, overlaid on CT scans of 864 × 864 resolution with 69 μm slice thickness.

The SPECT/CT scans of an irradiated liver showed considerably reduced uptake in the irradiated lobes compared to those spared from irradiation. This effect is especially striking in the cirrhotic DPPIV deficient C57Bl/6 mice and Rag2^−/−^γ(c)^−/−^ images as these animals are more radiosensitive compared to non-cirrhotic C57Bl/6 animals. We included a further example of an animal treated with radiation field to the median and left lobe showing persistently reduced tracer uptake 1 year after liver irradiation, indicating long-term RILD. Conversely, the SPECT/CT images of an age-matched control animal that was not irradiated shows uniform tracer uptake throughout the whole liver.

Taken together, the histopathological results showing DNA damage and the reduced liver function seen in the functional imaging studies indicate that the targeted hepatic irradiation technique induces region-specific liver damage limited to parts of the liver that were treated to a high doses of radiation.

## 4. Discussion

An experimental model of high-dose (50 Gy and 25 Gy) targeted liver irradiation is presented that leads to radiation liver injury as shown by functional SPECT/CT imaging. This correlates well with clinical data where the volume of liver receiving more than 30 Gy is associated with the risk of RILD [5]. This model provides a significant improvement over previous pre-clinical liver irradiation techniques that require either laparotomy or the use of larger rodent models such as rats [6,7,8,16].

Furthermore, using image-guided treatment planning and dose calculation as in our presented irradiation model makes it possible to deliver high doses of radiation to specific liver regions while avoiding dose limiting GI toxicity. This will allow for dose-response studies of RILD to be conducted with accurate dosimetry, precise localization and systematically varying the volume of irradiated liver tissue. As the role of SBRT in the management of liver cancer either from hepatocellular carcinoma or metastasis continues to evolve using multiple different doses and fractionations, this model can be used to test the biologic effects of these different dosing and fractionation regimens to determine their effects on liver injury as well as test potential mitigators for RILD and develop non-invasive methods to monitor liver injury following radiation therapy.

In contrast to the presented model, clinical liver irradiation is typically performed using a hypofractionated regimen of 3–5 fractions delivering a total dose of 50–60 Gy. Performing fractionated studies in animals will require careful positioning and setup to ensure reproducibility of the irradiated areas, as well as consideration of the varying biological effect compared to single fraction studies.

In addition to studying RILD and its potential mitigators, the presented model can be used for delivering preparative hepatic irradiation in experiments related to hepatocyte or sinusoidal endothelial cell transplantation [9,12]. In this setting the main improvement with the presented model will likely be the ability to visualize the radiation dose distribution on the CBCT images, so that different areas of the liver can be examined based on the dose delivered to that specific area.

Although ^99^Tc^m^-labelled Sulfur Colloid was used in this study other tracers such as ^99^Tc^m^-labelled diethylene triamine penta-acetate–galactosyl human serum albumin (^99^Tc^m^-GSA) has been used clinically and may offer complimentary advantages as it would not be dependent on liver blood flow and other biochemical processes. Furthermore, ^99^Tc^m^-mebrofenin could be used as another alternative to measure total or regional liver function.

There is of course a trade-off between translational relevance of the applied irradiation model and the time it takes to perform an experiment. Based on our experience it takes approximately 1 h per animal to perform the entire image-guided targeted liver irradiation procedure, including administration of contrast agent. However, with clinical management of primary liver tumors or liver metastases moving towards highly conformal techniques such as stereotactic body radiation therapy (SBRT) [17], animal models need to employ image guidance and conformal RT delivery to remain translationally relevant.

Using the present model, C57/Bl6 cirrhotic mice had a larger defect on SPECT imaging compared to non-cirrhotic C57/Bl6 mice. This is consistent with patient clinical patient data showing that patients with Child-Pugh B or C cirrhosis with primary HCC are more likely to experience liver toxicities as defined by worsening liver function [18,19,20]. While the radiosensitivity between human and mice livers may not be exactly the same, this is more likely related to a volume effect where radiation damage and necrosis can occur only after damage to a critical mass of tissue. The density of microvasculature and liver parenchyma in the mice livers is comparable to that of the human liver. This could explain why significantly higher doses of radiation are required to produce radiation induced changes in the smaller irradiated volume of the mouse liver.

## 5. Conclusions

Herein, we developed the presented hepatic irradiation model as a tool for researchers to study RILD or preparative liver irradiation in a translational setting that closely mimics that of clinical radiation oncology.
